# Shift Work and Occupational Accident Absence in Belgium: Findings from the Sixth European Working Condition Survey

**DOI:** 10.3390/ijerph15091811

**Published:** 2018-08-22

**Authors:** Hanan Alali, Lutgart Braeckman, Tanja Van Hecke, Magd Abdel Wahab

**Affiliations:** 1 Department of Electrical Energy, Metals, Mechanical Construction and Systems, Faculty of Engineering and Architecture, Ghent University, 9052 Zwijnaarde, Belgium; Hanan.Alali@ugent.be; 2 Department of Public Health, Faculty of Medicine and Health Sciences, Ghent University, 9000 Ghent, Belgium; Lutgart.Braeckman@UGent.be; 3 Department of Industrial Technology and Construction, Ghent University, 9000 Ghent, Belgium; Tanja.VanHecke@UGent.be; 4 Institute of Research and Development, Duy Tan University, Da Nang, Viet Nam; 5 Soete Laboratory, Faculty of Engineering and Architecture, Ghent University, 9052 Zwijnaarde, Belgium

**Keywords:** injuries, work accident, shift work

## Abstract

(1) Background: Irregular and non-standard work arrangements have become a serious determinant to the health and safety of workers. The aim of this study is to investigate the relationship between shift work and occupational accident absence. A representative Belgian sample considering several sociodemographic and work characteristics is used. (2) Methods: This study is based on the data of the sixth European Working Condition Survey (EWCS). The sample is restricted to 2169 respondents from Belgium. By using multivariate logistic regression modeling techniques and adjusting several confounders, the associations between shift work and occupational accident absence are studied. (3) Results: It is found that about 11.1% of the workers undergo an occupational accident absence. A multivariate regression model demonstrates an increased occupational accident absence risk for workers who have shift work (odds ratio, or OR, 1.92, 95% CI 1.06–3.46). Also, gender and biomechanical exposure were significantly associated with occupational accident absence ((OR 2.07, 95% CI 1.16–3.69) and (OR 2.03, 95% CI 1.14–3.63), respectively). No significant interaction effects are found with gender and age variables. 4) Conclusion: This study confirms that doing shift work is significantly associated with occupational accidents. In order to reduce the significance of occupational accidents, shift work should be limited through national-level policies.

## 1. Introduction

Following the definition of the International Labor Office (ILO), shift work is a method of work organization so that crews of workers succeed each other and work a certain schedule or shift at the same workstations to perform the same operations. The stipulated weekly hours for any worker can operate shorter than the undertaking. The term ‘shift work’ is often used when most of the working hours fall outside the standard daylight hours (between 7–8 a.m. and 5–6 p.m.), i.e. evening, night, or weekend shifts or when more than one work period is scheduled in a workday. Shift work can be considered as synonymous with odd, flexible, irregular, unusual, and non-standard working hours [1,2].

Shift working becomes a routine characteristic and is inevitable in the future for many reasons: (a) shift work is necessary for various sectors, e.g., such as transport, public health, communication, media, and internal and external security to provide 24 hours of work on-site; (b) shift work affects the return on capital investment; and (c) the dependency of modern industries on expensive machines and the continuity of their functioning is excessively necessary and cost-effective. Therefore, workers have to operate these machines round-the-clock [3].

In the past few decades, the population of shift workers, especially in developed and industrialized countries, has tremendously increased. Developing countries have experienced this phenomenon as well [4]. Almost 17% of workers across the European Union (EU) are involved in shift work, and gender difference is not an issue in this regard. Part-time workers do less shift work than full-time workers, and older workers perform shift work less often than younger workers. In 2010, almost 18% of the EU workers reported working a night shift, which is slightly less than reported in 1991 [5]. According to the sixth EWCS, about 21% of workers in the EU28 (Member States of the European Union) report working shifts in 2015, which represents a robust increase from the 17% registered in both 2005 and 2010 [6].

In the last decade, employers and legislators in Europe have become more and more aware that quality of employment, i.e., the wage, working hours, and other aspects related to the mutual agreement with social security and protection systems, is important to increase productivity and well-being [7]. However, the recent financial crisis, the application of new technologies, production, trade, the internationalization of investment, and the globalization of the labor market have resulted in a shift from the classical standard employment relationship into an increasing number of non-standard working time arrangements [8,9,10]. Therefore, the relationship between non-standard work arrangements, such as shift work, and health and safety measures, has become the issue of new investigations.

A large body of research showed that shift work was significantly related to work-related accidents compared to regular daytime work. Muñoz et al. (2014) reviewed 262 injury reports between 2007–2009 to describe occupational injuries among workers at a tertiary level hospital in south–central Chile [11]. They found that injuries occurred more frequently during the morning shift. Another review was conducted by Zhao et al. (2010) in which the target populations were health care workers who were involved in shift work [12]. Most of the findings in the literature have demonstrated that shift work was associated with a higher risk of sustaining work-related injuries in the health care sector. A study on offshore working time arrangements and a systematic review of studies examining offshore day/night shift patterns in relation to operational safety and individual health risks was presented by Parkes (2012) [13]. Offshore night work as a risk factor for impaired sleep, health problems, and injuries was identified by analyzing the survey data and accident/sickness records. The study of Wagstaff AS (2011) aimed at providing a systematic review of the empirical research regarding the relationship between accidents, shift work, and long work hours [14], which is most relevant to safety-critical activities, e.g., the transport and health sectors. Both long working hours and shift work present a well-documented and substantial effect on safety and shift work, including a substantial increased risk of injuries and accidents during nights. The review of Anderson et al. (2013) conducted in the rail industry in Australia concluded that fatigue cumulatively builds with each sequential shift, in which rest in between is unsuitable (less than 12 h); as a result, shift durations more than 12 h are associated with a double risk for accident and injury [15]. Limits on work and rest hours, including successive number of shifts and maximum shift period, should be defined by a regulatory system for fatigue arrangement within the rail industry. Santos (2011) concluded that the greatest risk of accidents occurred at night when compared to morning and noon shifts [16]. The increasing age of workers and the increase in the retirement age have given rise to considerable concern about the effect of age in the safety of shift workers [17]. The results indicated that there was clear evidence that the injury rates were higher at night than during daytime. Furthermore, the injury rates increased over successive night shifts faster than over successive day shifts. Also, it was concluded that it seemed possible that older workers might be at a greater risk than younger workers to injury and accident during night shifts [17].

In conclusion, research suggests that shift work is related to a higher rate of occupational injuries and accidents. However, this relationship has not yet been explored in a large-scale harmonized Belgian sample of the working population. Thus, this study aims to examine the associations between shift work and occupational accidents in a large dataset of Belgian employees, considering several demographic and work-related confounding factors.

## 2. Methods

### 2.1. Study Population

Since 1991, the European Working Conditions Survey (EWCS) has been used by Eurofound to monitor the working conditions in Europe. The aim of Eurofound is to evaluate the working conditions in European countries, identify groups at risk, and improve job quality. This periodically conducted survey makes use of face-to face questionnaires at the participants’ own home, and is considered as a main source of comparable data. To enable analysis over time, the sixth EWCS questionnaire contained questions from previous surveys, as well as new questions addressing emerging challenges and policy issues of interest. The sixth EWCS presented a diverse view of European workers over time across different countries, genders, age groups, and occupations. In 2015, almost 44,000 employed and self-employed workers were interviewed between February and September 2015 from 35 European countries (the 28 EU Member States, plus the candidate countries for EU membership, Montenegro, the former Yugoslav Republic of Macedonia, Serbia, Albania, Turkey, Switzerland, and Norway). The sample size ranged from 1000 to 3300 people per country, with three Member States (Belgium, Spain, and Slovenia) having supported a bigger sample size in their countries. Workers were asked a number of questions related to work and health, status of employment, health and safety, duration of working time, physical and psychosocial risk factors, learning and training, earnings and financial security, and work–life balance. Details on the questionnaire, sampling design, and methods are available elsewhere [18]. This study is based on the data of the sixth EWCS, and for the purpose of the current analysis, the analytical sample is restricted to a subgroup of 2169 employees in Belgium. Persons excluded are those who were unemployed, self-employed, or with an apprenticeship. The following question was used in the sixth EWCS to evaluate the outcome variable: “Can you indicate how many days were attributable to an accident at work, over the past 12 months, out of the days of absence?” The responses with zero days were classified as having no work accident, and the responses with more than one day of absence were classified as having an occupational accident. The following question was used to measure shift work: “Do you have shifts?” The response options were “yes” or ”no”.

### 2.2. Covariates

Studies in the literature have demonstrated that work injuries and accidents are known to be multifactorial. Work-related factors and sociodemographics play an essential role in their happening [19,20,21,22,23,24,25]. We selected and included these factors in our data analysis as potentially confounding variables. The confounding sociodemographic variables are sex, age in years, self-rated health level, and self-related educational level. Considered covariates that are related to work-related factors are contract type, long hours, multiple jobs, work experience, economic activity, company size, overall fatigue, risk information, sleep difficulties, and job exposures as: physical (PH), biological (BL), chemical (CH), and biomechanical (BM) exposure.

These covariates were taken into account in the multivariate analysis in order to oversee the potential confounding between the dependent variables and shift work.

The following question was used to assess self-rated health: “In general, how is your health?” The response options were very good, good, fair, bad, and very bad. This variable was treated as a dichotomous variable: “good” (very good and good) versus “bad” (fair, bad, and very bad). The highest level of education and successfully completed training of participants was also a question. The results were divided into four categories: (1) workers who had no education or completed primary school, (2) workers who completed lower or (3) upper secondary school, and (4) workers who additionally completed tertiary education. The following question defined the variable “contract type”: “What kind of employment contract do you have”? Those having “no contract, a temporary employment agency contract, a contract of limited duration, and an apprenticeship or other training scheme” were defined as having a precarious contract, and were compared to those who had a permanent contract. The long working hours were defined as 48 hours/week or more [6,26]. The following question was used to assess the variable “multiple jobs”: “Do you have any other paid job(s), besides your main paid job?” The response options were: (1) “no other paid job,” (2) “regular,” (3) “occasional,” and (4) “other.” Those who had (2) regular, (3) occasional, and (4) other paid jobs were categorized as category “yes”, whereas those with (1) no other paid job were categorized as “no”. The following question was used to evaluate work experience and included the number of working years: “How many years have you been in your organization or company?” A question regarding company size was included: “In total, how many people work at your workplace?” Responses were classified as (1) “small”: work alone, with two to four people, or with five to nine people; (2) “medium”: 10–49 people, 50–99 people; (3) “large”: 100–249 people, 250–499 people, and (4) “very large”: 500 people or over. The Statistical Classification of Economic Activities in the European Community, which is abbreviated as NACE, was used to code the economic activity of the company: (1) construction; (2) mining, manufacturing, quarrying, electricity, water supply, and gas; (3) agriculture, forestry, hunting, and fishing; and (4) services. The following question was used to measure the overall fatigue: “Did you suffer from overall fatigue over the past 12 months?” The response options were “yes” and “no”. The following questions were used to assess the sleep difficulties: “How often did you have any of the following sleep-related problems over the last 12 months? (a) difficulty falling asleep, (b) waking up repeatedly during the sleep, and (c) waking up with a feeling of exhaustion and fatigue”. The sleep difficulty variable was created from the sum of these three questions. The cases where all three questions were answered were used; otherwise, these cases were considered as missing values. This variable was also treated as a dichotomous variable with the response options “yes” and “no”. The following question was used to evaluate the risk information variable: “How well were you informed about health and safety risks related to the performance of your job?”. Finally, the job exposure variable was defined as: (1) PH exposure, (2) CH exposure, (3) BL exposure, and (4) BM exposure. This definition is according to the categories defined by Niedhammer et al. [26]. We introduced binary variables expressing PH, CH, BL, and BM exposures. The median of PH, CH, BL, and BM was used to dichotomize the answers. The internal consistency estimate of the reliability of test scores, Cronbach’s alpha, was almost 0.7 for PH, CH, BL, and BM exposures, which is an acceptable result.

(1) Physical exposure (PH) (three items) included being exposed at work to: (a) high temperatures that make you perspire even when not working; (b) noise so loud that you would have to raise your voice to talk to people; and/or (c) low temperatures whether indoors or outdoors. (2) Chemical exposure (CH) (four items) included being exposed at work to: (d) vapors such as solvents and thinners; (e) smoke, fumes (such as welding or exhaust fumes), powder, dust (such as wood dust or mineral dust), etc.; (f) handling or being in skin contact with chemical products or substances; and/or (g) tobacco smoke from other people. (3) Biological exposure (BL) (one item) included being exposed at work to: (h) being in direct contact with or handling materials that can be infectious, such as waste, laboratory materials, bodily fluids, etc. (4) Biomechanical exposure (BM) (five items) included being exposed at work to: (i) vibrations from hand tools, machinery, etc.; (j) lifting or moving people; (k) tiring or painful positions; (l) repetitive hand or arm movements; and/or (m) carrying or moving heavy loads.

### 2.3. Statistical Analysis

This statistical analysis is based on the data collected from the sixth EWCS. First, we computed the descriptive statistics for all of the variables, and then we carried out Chi-square tests to investigate whether or not potential risk factors (covariates) were univariately associated with the dependent variable, i.e. the occurrence of work-related accident absence. Finally, in order to explore the relation between shift work and work-related accident absence, we computed a series of binary regression models in three steps. (1) We calculated a crude model. (2) We entered into a second model the sociodemographic items such as gender, age, education level, and self-rated health. (3) We entered into a third model the sociodemographic items and work-related factors such as contract type, long hours, multiple jobs, work experience, economic activity, company size, overall fatigue, risk information, sleep difficulties, and exposures to PH, CH, BL, and BM. In all of the analyses, in order to prevent potentially important variables being rejected, we made adjustments for confounding variables, regardless of their univariate associations with the outcome. We screened models for multicollinearity between different independent variables according to the calculation of variance of inflation (VIF) factors, and we found no problems. We proceeded with the data and analyzed them using SPSS version 21, and we evaluated all of the models at a 0.05 significance level.

## 3. Results

Table 1 presents descriptive statistics of the studied sample. In the analyses, a total number of 2169 workers were considered. The studied population consisted of 1048 men (48.3%) and 1121 women (51.7%). The average age was 42.14 years (±11.41 SD), and the percentage of highly educated participants was 42.6%. The percentage of participants in good health was 79.6%. The percentage of workers who reported work-related accident absence during the past 12 months was 11.1%. About 14.2% of the participants had a precarious contract, whereas 6.9% worked long hours. A percentage of almost 7.4% of the participants had multiple jobs, and 18.1% had shift work. Almost a third of the participants, 38.9 %, reported overall fatigue, and 29.3% reported sleep difficulties.

The univariate association results from the Chi-square tests showed that workers doing shift work had more absence due to work accidents than non-shift workers. Concerning the relation between education level and absence due to work-related accidents, the univariate association showed that low-educated workers had more absence due to work accidents than highly educated ones, and the difference was statistically significant. A univariate association was observed between contract type and absence due to work accidents. Workers who declared having a precarious contract type were less absent from work due to work-related accidents than those declared having a permanent contract. Workers who were not well informed about the safety risk and health at work were more absent due to work accidents than their counterparts. Finally, regarding the job exposure variable, a univariate association was observed between all of the types of exposure as physical, chemical, biological, and biomechanical exposure, and absence due to work accidents. In contrast, no statistical significant differences were observed between men and women, nor were there any statistically significant difference observed between those who rated their health as good or bad in terms of being absent due to work accidents. Long hours, multiple jobs, economic activity, company size, overall fatigue, and sleep difficulty variables were not statistically significant (results are not shown).

From the multivariate logistic regression analysis, the odds ratios (OR) and 95% confidence intervals (95% CI) are summarized in Table 2. In the crude model, an increased injury risk was observed for shift workers (OR 2.30, 95% CI 1.53–3.43). In the second model that considered sociodemographic variables, shift work remained positively associated with work-related accident absence (OR 2.12, 95% CI 1.40–3.20). For low-educated workers, an increased occupational accident absence risk was observed (OR 2.45, 95% CI 1.09–5.50). In the third model that considered all of the work-related factors, shift work again remained significantly associated with occupational accident absence (OR 1.92, 95% CI 1.06–3.46). Also, from the sociodemographic variables, gender became positively associated with work-related accident absence (OR 2.07, 95% CI 1.16–3.69). Yet, the relationship between contract type, multiple jobs, working long hours, and work-related accident absence was not significant in the third model (OR 0.33, 95% CI 0.10–1.08), (OR 0.71, 95% CI 0.26–1.98), and (1.27, 95%CI 0.49–3.30), respectively. Among the exposure variables, only biomechanical exposure (BM) was positively significant with the outcome variable (OR 2.03, 95% CI 1.14–3.63).

## 4. Discussion

This study provides an overview on the relationship between shift work and work-related accident absence amongst Belgian workers. Generally, the results showed that shift work was significantly associated with occupational accidents absence. This finding is in line with previous work in this field [11,12,13,14,15,16,17,27]. It is explained that as shift work may contribute to the high risk of injuries, it may also cause lower levels of co-worker supervision and support during night work schedules [28,29,30,31,32]. Furthermore, shift work may be more stressful mentally, emotionally, and physically, and lead to a lack of concentration [33,34]. Another way to explain our finding is that shift work may disrupt the normal sleep styles and the body’s regular schedule, causing increased fatigue because of sleep disorder. Fatigue and sleepiness can lead to errors, injuries, work accidents, fatalities, absenteeism, and poor concentration. For example, up to 90% of shift workers reported regular fatigue and sleepiness at the workplace, and about 1/3 of shift workers were affected by insomnia [28]. Therefore, overall fatigue, stress, and sleeping difficulties are proposed as a plausible mechanism explaining the consistent relationship between shift work and occupational accidents.

The overrepresentation of shift workers in Belgium within work on unusual and flexible hours can be explained by Belgian machinery factories applying shift work. Plant and machine operators work in industrial factories. This industrial work environment contains heavy repetitive work. On the negative side of the heavy repetitive work cluster, workers have to work in a risky environment and have neither autonomy nor say at work. Lower educated employees more often perform heavy repetitive work. More than 50% of the Belgian workers were confronted with at least one uninteresting element of the work quality, and about 9% carried out the heavy repetitive work. In addition, night and shift work are avoided by part of the old workers, due to the unusual working times nature of this work such as eating and sleeping times are conflicting with the working rhythms and normal functioning of the body, are disconnected with the general pace of life in society [35]. Furthermore, Costa et al. [36,37] and Wedderburn [38] concluded that workers that have non-standard working times such as night and shift work, weekend, etc. present efforts higher than standard working hours, as they are opposing the standard human cycles, i.e., sleep–work–leisure cycles [36,37,38]. Recent report on the sixth EWCS reported that workers doing shift work are more likely to report that their safety and health is at risk and that work affects their health badly. In addition, it is less likely that they report a good fit between family and their work. Furthermore, it is more likely that they report working at high speed, feeling tired at the end of the working day, and not feeling paid appropriately for their achievements and efforts in their jobs [6]. All of these explanations can lead to a high risk of work-related accidents and absence among Belgian shift workers.

Another risk factor that is associated with occupational accident absence in this study was gender. In agreement with prior studies [6,20,39,40,41], men were considerably more likely than women to have an accident at work. As reported in the EU28, more than 2/3 (68.7%) of non-fatal accidents at work involved men. Part of the gender difference in relation to accidents at work may be attributed to the lower female presence in the labor market [39]. Another possibility that might explain this finding could be that men and women do not have the same working conditions or jobs. For example, women are employed in less dangerous jobs, such as service occupations or teaching. Also, few women work in high-risk jobs, such as in the construction sector [6].

Working conditions reported the presence of hazards during a usual working day e.g. exposure to smoke, fumes, dust, vapors, vibration, low and high temperatures, noise, uncomfortable or tiring positions, poisonous substances, the handling of heavy loads, and also doing repetitive tasks. Therefore, the relationship between exposure to chemical, physical, biomechanical, and biological exposures, and work-related accidents absence is of interest in this research work. We found that biomechanical exposure was the only statistically significant explanatory variable for having an occupational accident absence. Our results are in line with previous research, which concluded that exposure to vibrating machinery and the total number of job conditions predicted the odds of accidents and injuries [22,23].

A previous study on the associations between non-standard work arrangements and work-related accident absence in Belgium has been already published by our team [42]; however, it only examined the data from the EWCS 2010, and not from the EWCS 2015. In 2015, about 11.1% of Belgian workers were absent from work because of work-related accident absence, which was almost the same proportion as in 2010 (11.7%). Comparing the results of this study (using the sixth EWCS) to those from the fifth EWCS, we may conclude that Belgian shift workers and those exposed to biomechanical exposure are still at risk for work-related accidents absence. Thus, on the basis of these findings, we conclude that there are no major changes over time in Belgium regarding ameliorating the situation of shift workers. Furthermore, there is no hard date to conclude. A recent report on the sixth EWCS by Eurofound also showed an increase in shift and Sunday work percentages [43]. Overall, no statistically significant differences were observed for those having precarious work, multiple jobs, or long working hours, in relation to work-related accident absence between the fifth and the sixth EWCS. Despite the existence of legal practices in Belgium (suggesting that the conclusions reported in other studies might be different for Belgium) for ameliorating the situations of Belgian non-standard workers, still Belgian shift workers are at a higher risk of occupational accident and absence from the fifth and the sixth EWCS.

An additional point of interest has been put forward in earlier research reporting that shift workers, especially at night, often describe having sleep problems after work or being sleepy at work, and declared having more occupational injuries and accidents. This can be bad for their health, safety, and well-being [11,13,14,16,17].

In our study, we were unable to investigate only night or morning because the question used to measure the shift work variable was: “Do you have shifts?” The response options were “yes” and “no”. Therefore, we cannot investigate for those doing only night shift work.

A number of specific strengths of the current study should be mentioned. The participants of the present study were selected from a large harmonized sample of the Belgian working population. All of the responses were collected by interview at home, and the response rate for the sixth EWCS was about 43%, ranging from 11% in Sweden to 78% in Albania. For Belgium, the response rate was (36.3%).

Although the current study confirms evidence regarding the existing information about occupational accidents, there are several limitations. These limitations should be considered when interpreting the results. A possible shortcoming is that the results are based on self-reports. Furthermore, the respondents were asked only about their absence due to a work-related accident. However, they were not asked how many accidents they had during the last year, nor were they asked what the cause of the accident was or how severe it was. A reporting bias might be suspected related to common method variance. Yet, we noted that the questions were not specifically asking about the relationship between shift work and work-related accident absence, and were formulated in a general manner. Thus, we suppose that the common method variance bias can be limited. Another possible shortcoming of this study is that it was based on interviews, for which some information may not be truly representative. This is because some workers may have given more socially favorable answers by minimizing their number of absence due to work-related accidents. A final remark is that an association between two variables can be established due to the cross-sectional nature of this study. However, it is not possible to determine the causal link of this relationship.

## 5. Conclusions

In conclusion, the growing number of atypical working time and non-standard work arrangements becomes a serious threat to the health and safety of workers. In this study, we investigated the association of shift work with work-related accident absence. The findings from this study can be used as an important element in creating and implementing safety and health policies at a national (in this case Belgian) level. As shift work may affect the well-being of employees, it is important for employers to prevent damage to people and control risks efficiently. Employers might apply good practice guidelines to improve the shift-work schedule and the workplace environment by performing a method for the early reporting of problems associated with shift work and periodically review the performance of their shift workers. In addition, they could also develop clear procedures for managing shift work. So, at all levels of planning, limiting the risks of shift work needs to be considered by employers.

However, in order to prevent injuries, better employment arrangements and educational strategies are highly advised. In addition, to emphasize the importance of the development of more and better jobs, strategies should be put into place at the policy level. At the organizational and individual level, the implementation of an educational approach and more safety policies that aim at developing awareness about the harmful effects of shift work is recommended.

In summary, some studies suggest that shift work is associated with a higher rate of occupational accidents and injuries. However, this relationship has rarely been explored in a large harmonized sample of the Belgian working population. Therefore, this study is the first that aims to examine the associations between shift work and work-related accident absence in a large dataset of Belgian employees.

## Figures and Tables

**Table 1:** Characteristics of the study population (n = 2169).

Individual and Work-Related Factors	$\mathrm{Total}\;\mathrm{Study}\;\mathrm{Sample}:\;n\;{\left(\%\right)}^{\;a}$
Sociodemographic factors	
Gender n = (2169)	
Male	1048 (48.3)
Female	1121 (51.7)
Mean age/yr (SD)	42.14 (11.41)
Self-rated health n = (2168)	
Bad	442 (20.4)
Good	1726 (79.6)
Education level n = (2160)	
Primary level	86 (4.0)
Low secondary	312 (14.4)
High secondary	842 (39.0)
Tertiary level	920 (42.6)
Work-related factors	
Work-related accident absence n = (1207)	
No	1073 (88.9)
Yes	134 (11.1)
Contract type n = (2159)	
Precarious contract	306 (14.2)
Permanent contract	1853 (85.8)
Long hours n = (2169)	
Long hours	150 (6.9)
Normal hours	2019 (93.1)
Multiple jobs n = (2167)	
No	2007 (92.6)
Yes	160 (7.4)
Shift work n = (2164)	
No	1772 (81.9)
Yes	392 (18.1)
Mean work experience/year (SD)	11.68 (10.22)
Company size n = (1168)	
Small	163 (14.0)
Medium	508 (43.5)
Large	291 (24.9)
Very large	206 (17.6)
Economic activity n = (2151)	
Construction	91 (4.2)
Mining, quarrying, manufacturing, electricity, gas, and water	315 (14.6)
Agriculture, hunting, forestry, and fishing	16 (0.7)
Services	1729 (80.4)
Overall fatigue n = (2166)	
No	1323 (61.1)
Yes	843 (38.9)
Sleep difficulties n = (2169)	
No	1534 (70.7)
Yes	635 (29.3)
Risk information n = (2121)	
Well informed	1806 (85.1)
Not well informed	315 (14.9)
Physical exposure (PH) n = (2169)	
No	1830 (84.4)
Yes	339 (15.6)
Chemical exposure (CH) n = (2165)	
No	1930 (89.1)
Yes	235 (10.9)
Biological exposure (BL) n = (2167)	
No	1866 (86.1)
Yes	301 (13.9)
Biomechanical exposure (BM) n = (2167)	
No	1245 (57.5)
Yes	922 (42.5)

^a^ Calculated according to the percentage of the valid count.

**Table 2:** Odds ratios (OR) and 95% confidence intervals [95% CI] for work-related accident absence from multivariate logistic regression model with non-shift workers as the reference group.

Variables	Work-Related Accident Absence
*Model 1*	*Crude OR [95% CI]*
Shift work *(Yes vs. No ^c^)*	2.30 [1.53–3.43] *
*Model 2*	*Adjusted OR [95% CI]*
Shift work *(Yes vs. No ^c^)*	2.12 [1.40–3.20] *
Gender *(Men vs. Women ^c^)*	0.81 [0.56–1.18]
Age *(Continuous)*	0.99 [0.97–1.01]
Self-rated health *(Bad vs. Good ^c^)*	1.23 [0.81–1.86]
Education *(Low vs. High ^c^)*	2.45 [1.09–5.50] *
*Model 3*	*Adjusted OR [95% CI]*
Shift work *(Yes vs. No ^c^)*	1.92 [1.06–3.46] *
Gender *(Men vs. Women ^c^)*	2.07 [1.16–3.69] *
Age *(Continuous)*	0.98 [0.95–1.00]
Self-rated health *(Bad vs. Good ^c^)*	1.39 [0.78–2.49]
Education *(Low vs. High ^c^)*	1.64 [0.41–6.47]
Contract type *(Precarious vs. Permanent ^c^)*	0.33 [0.10–1.08]
Long hours *(Long vs. Normal ^c^)*	0.71 [0.26–1.98]
Multiple jobs *(Yes vs. No ^c^)*	1.27 [0.49–3.30]
Work experience (Continuous)	1.00 [0.99–1.00]
Company size *(Small vs. Large ^c^)*	1.84 [0.65–5.22]
Economic activity *(Construction vs. Services ^c^)*	0.99 [0.28–3.43]
Overall fatigue *(Yes vs. No ^c^)*	1.35 [0.76–2.41]
Sleep difficulties *(Yes vs. No ^c^)*	1.02 [0.56–1.86]
Risk information *(Not well informed vs. Well informed ^c^)*	1.50 [0.79–2.84]
Physical exposure (PH) *(Yes vs. No ^c^)*	1.88 [0.99–3.55]
Chemical exposure (CH) *(Yes vs. No ^c^)*	1.58 [0.75–3.35]
Biological exposure (BL) *(Yes vs. No ^c^)*	0.99 [0.48–2.04]
Biomechanical exposure (BM) *(Yes vs. No ^c^)*	2.03 [1.14–3.63] *

OR: Odds ratios, [95% CI]: 95% confidence interval; * Significant associations; ^c^ Reference category; Model 2: Adjusted for sociodemographic factors. Model 3: Adjusted for sociodemographic factors and, in addition, for all work-related factors. The proportion of the explained variance of the multivariate model is 17.1%, R^2^ = 0.171 (Nagelkerke R Square) for work-related accident absence.
